# Carbonate of Ammonia in Scarlet Fever

**Published:** 1873-09

**Authors:** G. J. S. Camden

**Affiliations:** Rhyl.


### Carbonate of Ammonia in Scarlet Fever.

By G. J. S. Camden, Esq., Rhyl.

The following treatment of scarlet fever has come down from master to pupil through four or five generations of medical men, —to myself from a partner I joined in 1828—therefore extending over a period of nearly 150 years. I was nearly losing a patient, when my partner told me if I persisted in treating scarlet fever *secundum artem* I should lose many. He then told me what he had been taught by his master, and had used for thirty years with the greatest success. I adopted his system, and am fully satisfied with the results. Never give emetics or aperients, nor bleed, nor use leeches, nor do anything to lower the power of life, but give ammon. carb. on the very onslaught of the disease, the earlier the better, when it will cut the disease short. I used it as follows:— ℞. Ammon. carb. gr. x. vel gr. xij., aquæ ʒ iv., ʒ vj., vel ʒ viij.— for 16 years and above. ℞. Ammon. carb. gr. viij. vel gr. x., aquæ ʒiv., ʒvj., vel ʒviij.—12 years to 16 years. ℞. Ammon. carb. gr. vj. vel gr. viij., aquæ ʒiv., ʒvj., vel ʒviij.—6 years to 12 years. ℞. Ammon. carb. gr. iv. vel gr. ʒvj., aquæ ʒij. vel ʒiij. —4 years to 6 years. ℞. Ammon. carb. gr. ij. vel iv., aquæ ʒ j. vel ij.—2 years to 4 years. Unless distilled water be used it must be cold boiled rain-water filtered, the dose to be taken every two, four, or six hours, according to the severity of the throat symptoms; the quantity of water to be regulated on the same principle. The worse the throat the stronger the dose of ammonia, the smaller quantity of water, and to be given most frequently. The choking from the ammonia is instantly relieved by a small quantity of cold water, but if done without the better. If the power of life is at a low ebb, wine or teaspoonful of brandy, and the same of water between each dose, and beware of aperients. I have waited five or six days. The foregoing prescriptions I sent to a lady in Ireland, who had seen the effect in eleven cases, in her own house. In the original treatment in cases in which the tonsils had become gangrenous, the following was used as a gargle:—℞. Rad. pyrethri ℥iij., aquæ ℥xvj., decoque ad ℥x. et cola; adde syrup. rheados ℥ij.—M. Gargar. sæpe utend. My partner used it whilst with me but once; I never used it, though I had one extremely severe case with gangrenous throat, through the nurse's negligence. There were twenty-two patients in the house—a school—and none died. I only used the ammonia and the brandy. In each case the child recovered. I never used leeches but once—the child being delirious —and then put on only two, and as soon as they came off stopped the bleeding. In my severe case ascites supervened, which nothing relieved. After several months in dread of paracentesis the umbilicus ulcerated, the cavity emptied, the child recovered, and grew a fine young woman. One great essential is the room kept cool and well ventilated.

Some few of my medical brethren have followed the treatment on my telling them, and were as much satisfied as myself; but most are incredulous. I never lost a dozen patients from scarlet fever in the course of twenty-five years, though I lost two in forty-eight hours in one house; but that was the abominable situation of it—the corner of a small wood into which the drainage from a large farm yard ran in close proximity.

About the year 1838 (I think) there was a letter in the *Lancet* in which the use of ammon. carb. in scarlet fever was mentioned as a new discovery by a German M.D. Since then two letters have appeared in the *Times* from Dr. C. Witt—one on December 1, 1858, the other I forgot when. Of diphtheria I know nothing, but believe it to be only another phase of scarlet fever. Of the sequelæ you have less after the ammonia treatment, having seen but little, and, should anasarca supervene, it will readily yield, as I have of late years found (with alternate doses of quinine as a tonic), to liberal doses of potass. bicarb. (Howard's) with potass. nitrat. taken in a large quantity of water. The potass. nitrat. I use is to be obtained only at powder makers'. It has been melted by heat and kept so for two or three days, so that all water of crystalization is driven off. I mention this as I have always used it, and fancy I should not get a similar effect from any other. The sudden retrocession of the eruption I never knew to be of consequence; but the most severe and frequently fatal cases are usually those in which the eruption does not appear, and these cases are more frequent than is supposed, and are not suspected till too late. To my eye there is such a peculiar appearance of the throat it cannot be mistaken. —*Medical Times and Gazette.—Braithwaite's.*

